# Trophoblastic tumor in perimenopausal women: A case report and literature review

**DOI:** 10.1016/j.ijscr.2025.111130

**Published:** 2025-03-09

**Authors:** Ghaddab Imen, Medemagh Malek, Toumi Dhekra, Njima Manel, Jmaa Yosra, Hajji Ahmed

**Affiliations:** a Department of Gynecology and Obstetrics, CHU Fattoma Bourguiba, University of Monastir, Tunisia; b Laboratory of Histology-Pathology, CHU Fattoma Bourguiba, University of Monastir, Tunisia; c Department of Gynecology and Obstetrics, Hadj Ali Soua University Hospital, Monastir, Tunisia

**Keywords:** Gestational trophoblastic disease, Placenta diseases, Trophoblastic tumor, Placental site, Pathology, Case report

## Abstract

**Background and importance:**

Placental site trophoblastic tumor (PSTT) a rare form of gestational trophoblastic disease, originates from intermediate trophoblastic cells and presents with nonspecific symptoms, complicating diagnosis. PSTT primarily affects women of childbearing age, but occurrences in perimenopausal women are exceptionally rare.

**Case presentation:**

We report a case of a 54-year-old perimenopausal woman presenting with a two-month history of abnormal uterine bleeding. Clinical and imaging evaluations revealed an enlarged uterus and an intracavitary mass. Elevated β-hCG levels prompted suspicion of a trophoblastic tumor. Histopathological examination confirmed PSTT. The patient underwent total hysterectomy with bilateral salpingo-oophorectomy as definitive treatment. Postoperative outcomes were favorable, with normalization of β-hCG levels and no evidence of recurrence during two years of follow-up. Immunohistochemical staining for HPL and cytokeratin further confirmed the diagnosis.

**Clinical discussion:**

This case highlights the importance of integrating clinical, imaging, and histopathological findings for the accurate diagnosis of PSTT. Unlike other gestational trophoblastic neoplasms, PSTT is characterized by low sensitivity to chemotherapy, making surgical management the cornerstone of treatment. Long-term follow-up is essential to monitor for potential recurrence.

**Conclusion:**

PSTT is a rare and diagnostically challenging condition, particularly in atypical presentations such as in perimenopausal women. Early and accurate diagnosis, followed by surgical intervention, is critical for favorable outcomes. This case emphasizes the need for heightened clinical awareness and a multidisciplinary approach in managing such rare conditions.

## Highlights

• Rare Diagnosis: PSTT is extremely rare in perimenopausal women.
• Unusual Onset: Occurred 12 years post-pregnancy, an atypical timeline.
• Diagnostic Challenge: Nonspecific symptoms require clinical, imaging, and histopathological correlation.
• Surgical Approach: Hysterectomy with salpingo-oophorectomy is key due to poor chemo response.
• Positive Outcome: Early diagnosis and surgery led to sustained remission for two years.
• Clinical Impact: Highlights the need for multidisciplinary care and long-term follow-up.

## 1 Introduction

The placental site trophoblastic tumor (PSTT) is a rare type of gestational trophoblastic disease (GTD), characterized by a slow-growing malignant tumor that originates from intermediate trophoblastic cells [1]. PSTT can occur after a normal pregnancy, abortion, term delivery, ectopic pregnancy, or molar pregnancy. Unlike other forms of GTD, PSTT is characterized by low beta-hCG levels because it is a neoplastic proliferation of intermediate trophoblastic cells [2,3]. The most common presenting symptoms of PSTT are vaginal bleeding and amenorrhea. The diagnosis is confirmed by dilation and curettage, and hysterectomy, but meticulous evaluation for metastasis is mandatory. For the PSTT patient, surgery is the primary treatment of choice. We report an unusual case, following the SCARE guidelines [4], to highlight its distinctive features.

## 2 Case presentation

**Fig. 1:**
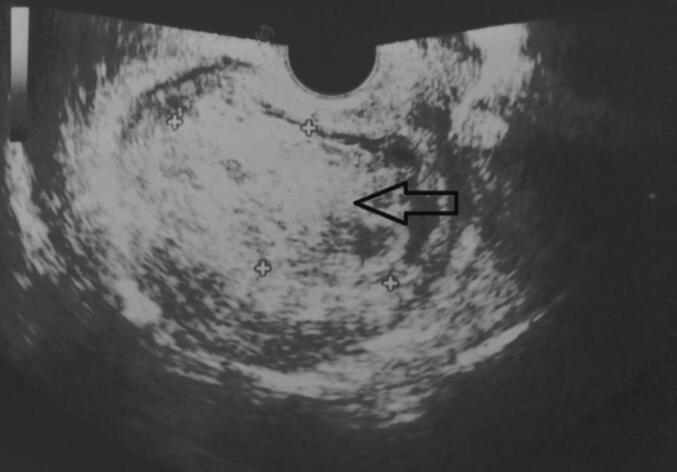
Echogenic tumor that occupies the uterine cavity (arrow) with infiltration of the myometrium.

**Fig. 2:**
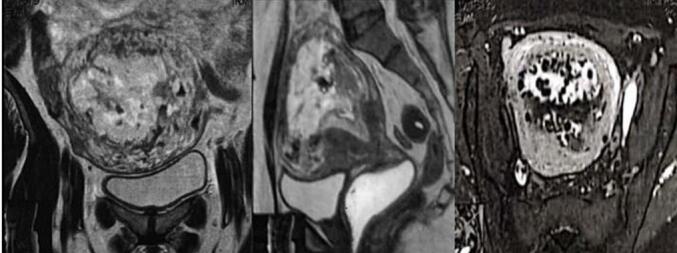
Magnetic resonance imaging view showing a poorly defined intracavitary mass of heterogeneous signal associated with cervicoisthmic endometrial thickening.

**Fig. 3:**
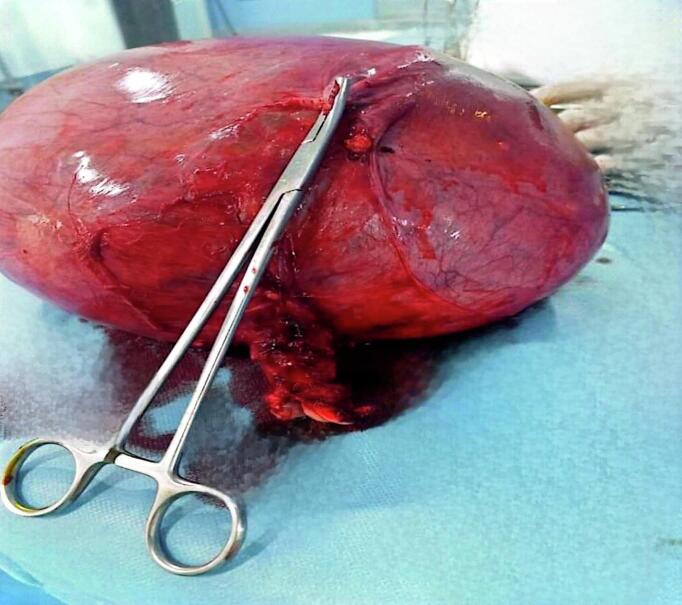
Macroscopic appearance of the enlarged uterus.

**Fig. 4:**
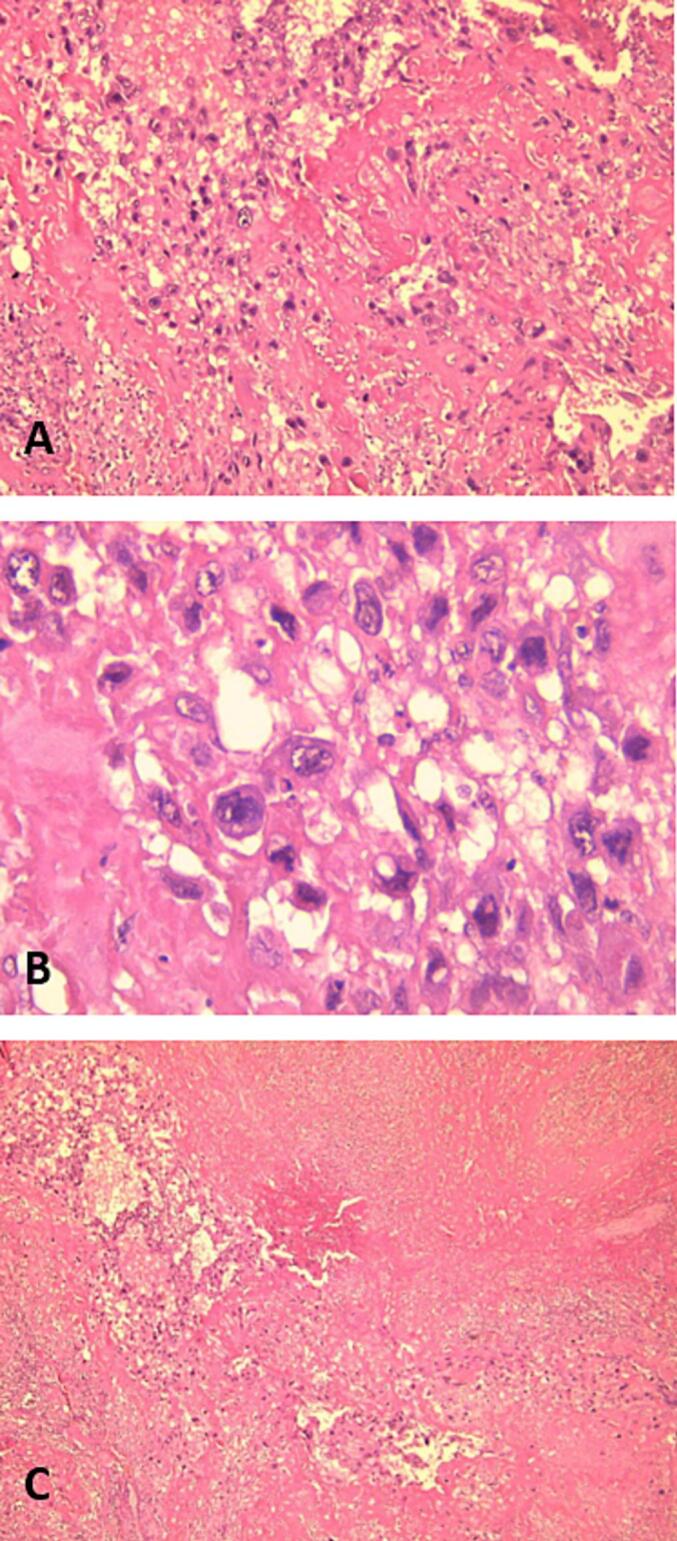
(A) Clusters of cytotrophoblastic tumor cells with abundant eosinophilic or amphophilic cytoplasm and very atypical nucleolated nuclei (HEx100), (B) Clusters of cytotrophoblastic tumor cells with abundant eosinophilic or amphophilic cytoplasm and very atypical nucleolated nuclei HEx400, and (C) The tumor is largely necrotic and dissociated by hemorrhage (HEx40).

A 54-year-old woman, gravida 4, with her last pregnancy occurring 12 years prior, presented with abnormal vaginal bleeding that persisted for two months. Her postpartum course following her previous pregnancies was unremarkable, with no history of postpartum hemorrhage or puerperal fever. The patient had no significant medical history, nor a personal or family history of GTD. On presentation to the emergency department, a physical examination revealed no abnormalities of the heart, lungs, or extremities. Gynecological examination identified a normal vulva, minimal vaginal bleeding, and an enlarged uterus approximately the size of an 8-week pregnancy. The uterus was soft on palpation with mild tenderness in the adnexal region. The laboratory evaluation showed an elevated β-hCG level of 199,217 mIU/mL. Transvaginal ultrasound revealed an intracavitary mass measuring 8 × 4 cm, characterized by chaotic vascularization in color Doppler (Fig. 1). Pelvic magnetic resonance imaging (MRI) confirmed the presence of a poorly defined heterogeneous intrauterine mass measuring 96 mm along its longest axis. The lesion invaded both the cervical stroma and the myometrium (Fig. 2). The imaging findings, combined with the markedly elevated β-hCG level, raised suspicion of an invasive trophoblastic tumor. An endometrial biopsy was performed. Histopathological analysis revealed sheets of intermediate trophoblastic cells infiltrating the myometrium. Immunohistochemical staining was positive for human placental lactogen (HPL) and cytokeratin, confirming the diagnosis of PSTT. Considering the localized disease and the nature of the tumor, a total hysterectomy with bilateral salpingo-oophorectomy was recommended as the definitive treatment. The procedure was performed successfully without intraoperative complications, and no residual tumor was identified (Fig. 3). The postoperative course was uneventful, with normalization of β-hCG levels observed during follow-up. Pathological examination confirmed the findings from the curettage specimen (Fig. 4), revealing a proliferation of monomorphic intermediate trophoblastic cells with moderate to marked nuclear atypia. The nuclei were irregular, hyperchromatic, and exhibited prominent nucleoli. The cytoplasm was eosinophilic, and there was extensive myometrial invasion. Areas of significant necrosis, hemorrhage, and vascular invasion were also observed. Immunohistochemical staining demonstrated strong positivity for human placental lactogen (HPL) and cytokeratin, further supporting the diagnosis of PSTT. Postoperatively, patient's hCG level rapidly declined to undetectable levels, consistent with successful tumor resection. The patient was monitored with regular follow-up, including hCG testing every 3 months and imaging every 6 months. At the two-year follow-up, the patient remained disease-free, with no evidence of recurrence on imaging or clinical symptoms. This outcome is consistent with a good prognosis for patients with localized PSTT treated with complete surgical resection.

## 3 Discussion

PSTT is a rare subtype of GTD. It accounts for less than 1 % of all cases [5,6]. This tumor was first described by Kurman and Scully in 1976 as pseudotumor trophoblasticum due to the benign nature of the lesion. Further evidence of its malignant behavior led to the nomenclature being revised to PSTT in 1981, and it has since been adopted by the World Health Organization [7]. PSTT often affects women of childbearing age. There is usually less than 2 years between the previous pregnancy and the onset of the tumor [8]. The particularity of our case lies in the fact that the tumor manifested 12 years from the last pregnancy and in a perimenopausal woman, such clinical presentation was rare in the literature. The clinical presentation of PSTT is often misleading, as it is nonspecific and can resemble both benign and malignant gynecological conditions. Patients usually present with amenorrhea or abnormal uterine bleeding. Examination reveals that the uterus is uniformly or irregularly enlarged. The vast majority of PSTTs appear as benign lesions, most of which develop inside the uterus [9].

Therefore, a comprehensive differential diagnosis is essential to guide appropriate management. Differential diagnosis includes choriocarcinoma, which is highly vascular and presents with much higher β-hCG levels, and invasive mole, which retains hydropic villi and has a different histopathological profile. Endometrial adenocarcinoma may also be considered but lacks trophoblastic markers such as HPL.

In most patients with PSTT, serum β-hCG levels are not elevated, which is different from other forms of gestational trophoblastic disease. PSTT typically produces lower β-hCG levels than choriocarcinoma or invasive mole because it originates from intermediate trophoblastic cells, which have limited β-hCG secretion. However, in some cases, particularly those with extensive disease, β-hCG may be markedly elevated, as observed in our patient. The expression of HPL is usually elevated in histological sections. Furthermore, serum β-hCG levels are not associated with malignant behavior. This means that serum β-hCG levels cannot be considered an accurate indicator of tumor burden and are rarely used to assess prognosis [4,10].

GTD, including PSTT, is primarily diagnosed by transvaginal ultrasound, which can detect features such as echogenic and vascular masses, sometimes extending into the myometrium. The ultrasound findings vary, with some cases showing a thickened endometrium or masses that contain cystic clusters. Magnetic resonance imaging further helps to assess myometrial involvement, with tumors appearing distinct in T1 and T2 weighted images. A multimodal imaging approach is essential to establish the extent of disease involvement and guide surgical planning. For staging and detecting metastasis, especially in the lungs, CT is indicated [11]. Unlike other trophoblastic tumors, PSTT is relatively insensitive to chemotherapy, and surgery is the main therapeutic approach in patients whose disease is confined to the uterus [12]. In the case of metastatic disease at the time of diagnosis, patients cannot be cured by surgery alone and require combined chemotherapy [13,14].

However, PSTT has been reported to recur even after prolonged disease-free intervals. Long-term follow-up beyond two years is recommended, including periodic β-hCG monitoring and imaging. This highlights the necessity of sustained surveillance, as late recurrences have been documented in the literature.

## 4 Conclusion

PSTT is a rare and diverse form of GTD, often confined to the uterus but presenting significant diagnostic and therapeutic challenges. Accurate diagnosis is essential, as PSTT differs from other gestational trophoblastic neoplasms, and delays can adversely affect management and outcomes. This case emphasizes the importance of integrating clinical, laboratory, imaging, and histopathological findings for prompt identification. Although localized cases, such as the one presented, are generally successfully managed with hysterectomy, metastatic cases may require chemotherapy. In this patient, the timely surgical intervention resulted in normalization of β-hCG levels and sustained remission over a two-year follow-up period, highlighting the potential for an excellent prognosis with early diagnosis and appropriate treatment.

Recognizing the unique features of PSTT is crucial to differentiate it from other gestational trophoblastic diseases and ensure timely intervention.

Furthermore, given the potential for late recurrence, long-term surveillance remains critical to detect any recurrence and ensure continued favorable outcomes.

## Authors' contributions

All authors participated in the treatment of the patients, writing and approving the manuscript.

## Consent

Written informed consent was obtained from the patient's parents/legal guardian for publication and any accompanying images. A copy of the written consent is available for review by the Editor-in-Chief of this journal on request.

## Patient consent

Written informed consent was obtained from the patient to publish this case report and accompanying images. On request, a copy of the written consent is available for review by the Editor-in-Chief of this journal.

## Ethical approval

All procedures performed in studies involving human participants were by the ethical standards of the institutional and/or national research committee and with the 1964 Helsinki Declaration and its later amendments or comparable ethical standards. Ethical clearance was not necessary as the format of this paper is a case report.

## Guarantor

Ghaddab Imen.

## Sources of funding

This research did not receive specific grants from the public, commercial or not-for-profit sectors.

## Funding

This research did not receive specific grants from the public, commercial or not-for-profit sectors.

## Declaration of competing interest

There is no conflict of interest to disclose.
